# Sustainable lactic acid production from agricultural waste: a review of current techniques, challenges and future directions

**DOI:** 10.1186/s40643-025-00923-3

**Published:** 2025-07-29

**Authors:** Nurasyikin Abdul Rahman, Zainab Ngaini, Saba Farooq, Sabrina Chua Ai Ling, Puteri Nabilah Jefree Shahren

**Affiliations:** 1 Faculty of Resource Science and Technology, Universiti Malaysia Sarawak, Kota Samarahan, Sarawak, 94300 Malaysia; 2 Malaysian Pepper Board, 93916 Kuching, Sarawak, Malaysia; 3 Department of Basic & Applied Chemistry, Faculty of Science and Technology, University of Central Punjab, Lahore, 54000 Pakistan

**Keywords:** Biodegradation, Food, Green, Lignocellulose, Synthesis

## Abstract

The increasing demand for lactic acid (LA), driven by its applications in biodegradable plastics, food additives, pharmaceuticals, and cosmetics, necessitates the development of sustainable production methods. Traditional processes often rely on food-based feedstock, aggravating food security concerns. This review addresses the critical gap in utilising agricultural waste as a non-food feedstock for LA production, thereby reducing waste and promoting a circular economy. Current techniques are thoroughly explored, including fed-batch, batch, and continuous fermentation procedures, as well as the utilisation of various microbial strains, including *Lactobacillus* species. Recent developments in genetic engineering and innovative fermentation techniques are discussed, alongside challenges such as substrate variability and contamination. By highlighting the potential of agricultural residues, such as sugarcane bagasse, rice husk and corn stover, this review provides a roadmap for future research and industrial applications to enhance sustainability and efficiency in LA production.

**Supplementary Information:**

The online version contains supplementary material available at 10.1186/s40643-025-00923-3.

## Introduction

**Fig. 1:**
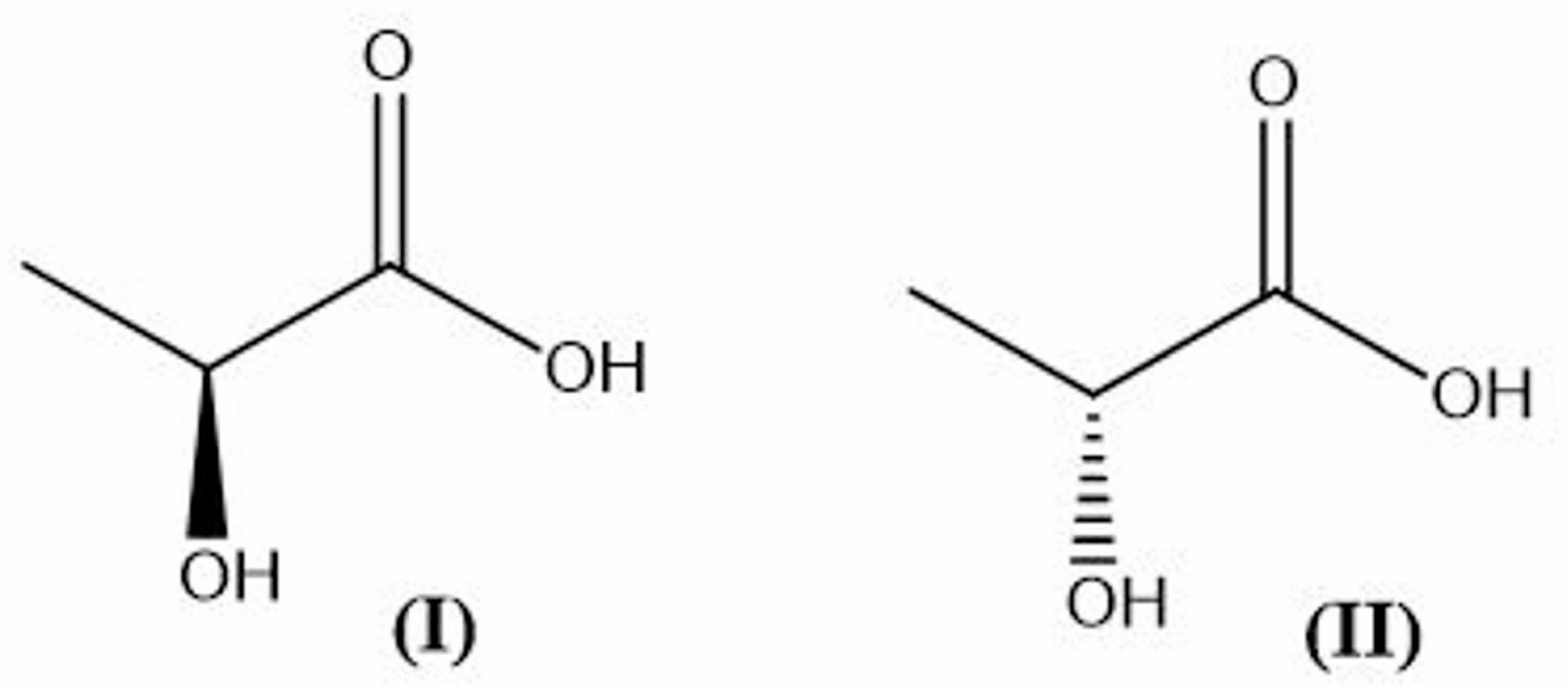
Chemical structure of L-LA **(I)** and D-LA conformation **(II)**

The increasing demand for sustainable and eco-friendly products has sparked significant interest in producing LA from renewable resources. LA, which exists in two forms (i.e., laevorotatory (L) and dextrorotatory (D) (Din et al. 2021) (Fig. 1), is a versatile organic acid (Castillo Martinez et al. 2013; Yankov 2022; Ajala et al. 2020; Thygesen et al. 2021) and widely used in various industries, including food, pharmaceuticals, and biodegradable plastics due to its ability to be easily absorbed by the body (Remund et al. 2023; Rodrigues et al. 2017; Mora-Villalobos et al. 2020). LA is a naturally occurring compound produced through the fermentation of carbohydrates found in plant-based and dairy waste, molasses, starchy materials, and lignocellulosic biomass (Abedi and Hashemi 2020). The high demand for LA is driven by its numerous benefits across various industries. In the food and beverage sector, LA serves as a natural preservative and flavour enhancer (Zapaśnik et al. 2022), particularly in fermented products, such as yoghurt and cheese. Its use in cosmetics as a natural exfoliant has also surged due to consumer preference for organic skincare products (Algiert-Zielińska et al. 2019; Feng et al. 2024).

Additionally, LA plays a significant role in pharmaceuticals as an excipient and in biodegradable implants. It is also a key precursor for polylactic acid (PLA), a biodegradable plastic, which supports global efforts to reduce environmental impact. Furthermore, its uses in agriculture as a biostimulant and in animal feed as an acidifier further increase its demand (Macias-Benitez et al. 2020; Raman et al. 2022). Overall, its versatility, natural origin and eco-friendly applications enhance its popularity and demand worldwide.

Typically, LA can be sustainably produced from agricultural waste. This innovative approach utilises crop residues, such as wheat straw and corn stover, as feedstock for fermentation processes. By employing microorganisms like *Rhizopus oryzae*, these carbon-rich agricultural byproducts can be efficiently converted into L-LA. This approach not only reduces waste but also offers an eco-friendly alternative to traditional production methods. The LA generated by this process can be utilised in the production of biodegradable plastics, food additives, and pharmaceuticals, thereby supporting the advancement of a circular economy and promoting sustainable practices. Traditional LA production methods rely heavily on petrochemical processes, which are both environmentally detrimental and unsustainable in the long term. Consequently, there is a growing need to explore alternative production methods that utilise agricultural waste. The readily available and renewable resource offers green and biodegradable production methods, presenting significant commercial opportunities (Alves de Oliveira et al. 2018; Yin et al. 2023). The traditional petrochemical-based methods for utilising agricultural waste for LA production represent a significant step toward sustainability (Ponce et al. 2025; Leung et al. 2025). Petrochemical-derived processes contribute to high carbon emissions, resource depletion, and environmental pollution, whereas agricultural waste-based methods promote renewability, lower emissions and reduced waste generation (Drishya et al. 2025). By repurposing organic byproducts, this approach minimises ecological harm while supporting a circular economy. The shift not only enhances environmental responsibility but also improves economic feasibility and long-term sustainability in LA production (Singhvi et al. 2018).

Despite the potential benefits, such as immunological tolerance, memory formation, wound healing, energy regulation, and cancer growth (Algiert-Zielińska et al. 2019; Sun et al. 2019; Li et al. 2022), the utilisation of agricultural waste for LA production faces several challenges. Current research primarily focuses on optimising fermentation processes and microbial strains to enhance LA yield. Microbial fermentation is preferred because it ensures high stereochemical control, reduces environmental impact and offers a sustainable, renewable approach to LA production (Pérez-Alvarado et al. 2022). A techno-economic analysis of LA production from lignocellulosic biomass (corn stover and miscanthus) at a 100,000-ton annual scale highlights key cost factors including raw materials, energy, capital investment, and process efficiencies. Among bacterial, fungal, and yeast fermentation pathways, genetically engineered yeast demonstrated the lowest production costs (USD 993–1123/ton), primarily due to enhanced sugar conversion and simplified downstream processing (Manandhar and Shah 2023). However, a significant gap remains in understanding the variability of agricultural substrates and their impact on fermentation efficiency.

Additionally, the issue of contamination and the economic feasibility of large-scale production remain to be fully addressed. Despite the potential of agricultural waste as a sustainable carbon source for LA production (Abdel-Rahman et al. 2013; Manandhar and Shah 2023), the efficiency and scalability of current fermentation processes remain limited due to challenges such as substrate variability, microbial contamination and suboptimal fermentation conditions. There is a lack of comprehensive studies addressing the optimisation of fermentation parameters and the development of robust microbial strains capable of efficiently converting diverse agricultural wastes into LA. Additionally, the integration of advanced pretreatment methods to improve the bioavailability of fermentable sugars derived from lignocellulosic biomass has not been extensively explored.

This review aims to bridge these gaps by comprehensively discussing the current techniques, challenges, and future directions in LA production from agricultural waste. A comprehensive discussion of the sources of agricultural waste (e.g., sugarcane bagasse, rice husk and corn stover), as well as fermentation processes utilising various fermentation techniques (e.g., batch, fed-batch and continuous fermentation) and types of microbial strains (e.g., Lactobacillus species) is explored. Challenges in the process (e.g., substrate variability and contamination, and potential solutions) and recent advances in LA production are also highlighted, focusing on recent advancements in the field, such as genetic engineering of microorganisms and novel fermentation strategies, as well as the applications including biodegradable plastics, food additives, cosmetics, medical and pharmaceuticals.

## General synthesis of LA

**Fig. 2:**
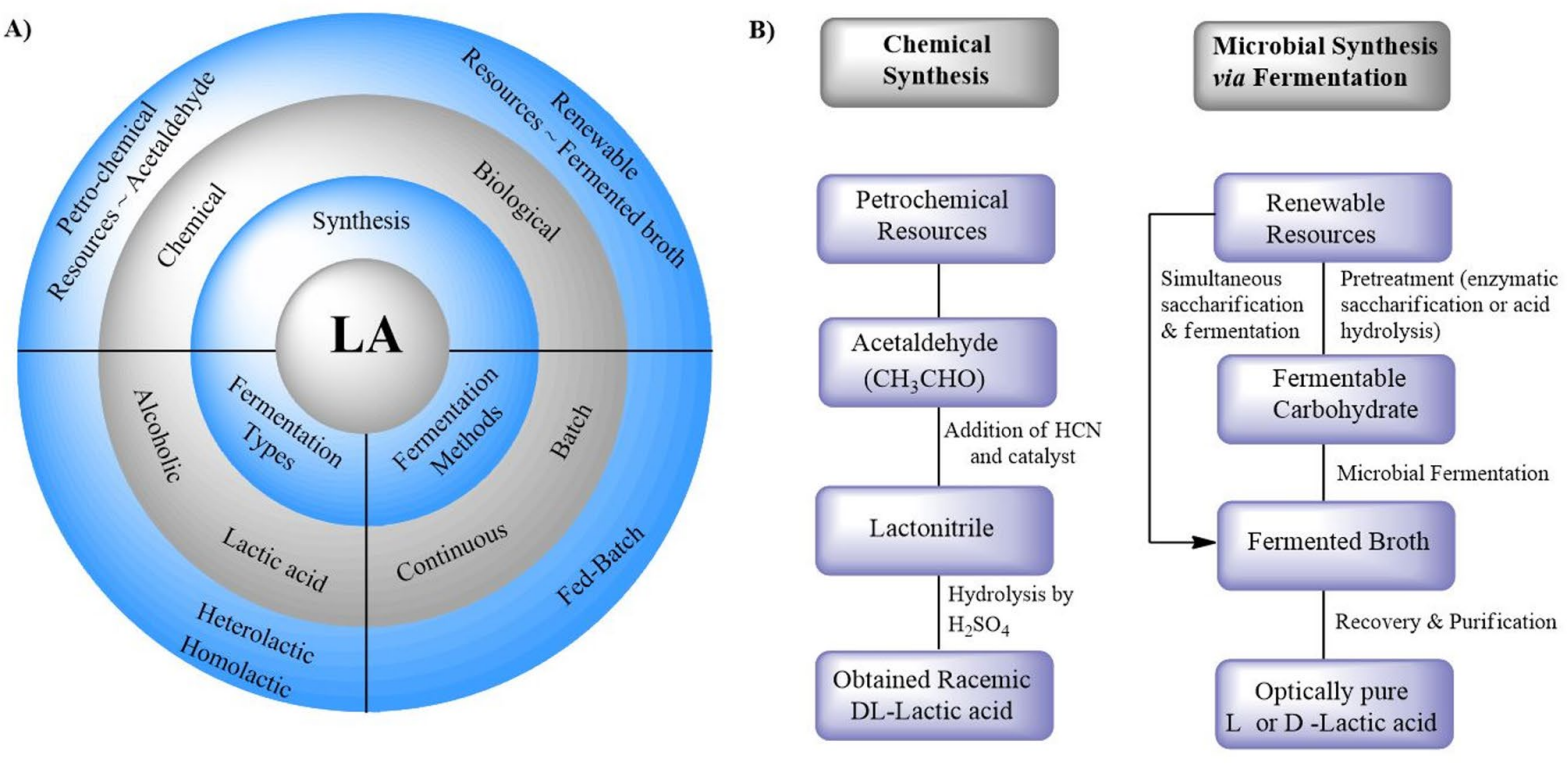
General synthesis of lactic acid and fermentation types **(A)** and chemical and microbial synthesis **(B)**

LA production can be achieved through two primary methods: chemical synthesis and microbial fermentation. While chemical synthesis involves petrochemical resources, microbial fermentation utilises renewable biomass, making it a more sustainable and environmentally friendly approach. The general synthesis of LA, types of fermentation and the chemical and microbial synthesis are depicted in Fig. 2.

### Chemical synthesis

The chemical synthesis of LA primarily involves the hydrolysis of lactonitrile using strong acids, such as sulfuric or hydrochloric acid, resulting in a racemic mixture of D- and L-LA enantiomers. This process lacks stereochemical control, leading to the formation of a non-chiral product. Lactonitrile is synthesised from acetaldehyde and hydrogen cyanide under high pressure, followed by distillation for purification. Subsequent hydrolysis of lactonitrile yields LA, which can be further processed into methyl lactate through esterification with methanol(Rawoof et al. 2021). A racemic mixture of DL-LA results from the chemical production process (Ghaffar et al. 2014; Ojo and de Smidt 2023). Other potential chemical synthesis routes for LA include base-catalysed sugar degradation, oxidation of propylene glycol, the reaction of acetaldehyde, carbon monoxide, and water at high temperatures and pressures, hydrolysis of chloropropionic acid and nitric acid oxidation of propylene (Madhavan Nampoothiri et al. 2010; Narayanan et al. 2004).

### Microbial fermentation

LA can also be produced through fermentation using selected microorganisms (Abedi and Hashemi 2020). Microbial fermentation using renewable green energy sources, such as lignocellulosic biomass, is a viable approach for LA production (Din et al. 2021; Nwamba et al. 2021). It is considered the most suitable and well-accepted process for the preparation of LA due to its cost-effectiveness (Abedi and Hashemi 2020; Yankov 2022). Since microbial carbohydrate fermentation is more practical (both chemically and financially) than the chemical method and allows for the manufacture of optically pure LA, it is the basis for the current industrial production of LA (Inkinen et al. 2011). Microbial fermentation commonly yields high optical purity L (+) or D (−) LA, while chemical methods yield a racemic form of D/L (±) LA (Rawoof et al. 2021). Microbial fermentation can yield D- or L-LA isomers based on the specific type of microorganism. It also offers an opportunity to enhance environmentally friendly production techniques and use cost-effective, low-energy substrates (Ahmad et al. 2020). LA production can be enhanced by utilising renewable feedstocks such as agricultural waste and lignocellulosic biomass, which have a high carbohydrate content and offer an abundant and low-cost alternative to traditional raw materials. Agricultural waste, such as sugarcane bagasse, corn stover and wastepaper, can be converted into fermentable sugars for microbial fermentation. However, challenges such as low yield and high production costs remain.

Utilising microbial consortia and rice straw enables LA production with high yields and cost-effectiveness. Microbial fermentation is a process in which microorganisms convert carbohydrates into LA. Many factors, such as microbial strains, substrates and fermentation techniques, influence the process of achieving efficient and cost-effective fermentation (Kacaribu and Darwin 2024) (Fig. 2). Selecting an appropriate bacterial strain is crucial as it influences key factors such as productivity, yield, purity and nutritional requirements (Rodrigues et al. 2017). Several challenges could hinder LA production from bacterial sources. These include low yields attributable to byproduct formation, the necessity for nutrient-rich media, a heightened risk of cell lysis and the requirement for mixed strains to develop phage-resistant populations to mitigate bacteriophage infections.

Fermentation is further classified into different types based on alcoholic and non-alcoholic (i.e., homolactic and heterolactic) strategies (Fig. 2(A). Homolactic fermentation is the production of two LA molecules from one glucose molecule. Heterolactic fermentation is the LA and side products (i.e., ethanol and carbon dioxide) from one glucose molecule.

Different fermentation techniques have been reported, including batch, fed-batch and continuous fermentation (Bolmanis et al. 2023). These techniques are essential for enhancing the nutritional value, sensory properties and shelf life of LA products. Batch fermentation is the simplest method, where all ingredients are added at the beginning, and the process runs until completion. This method, which involves adding substrates and nutrients to the bioreactor at the start, offers high yields and lower contamination levels. However, it also has disadvantages such as substrate inhibition and the need for frequent restarts. Fed-batch fermentation involves the gradual addition of substrates, allowing for better control over nutrient levels and reducing the risk of substrate inhibition. All techniques require consistent optimisation, such as high cell density cultures, co-cultures, fed-batch fermentation and neutralising agents (Huang et al. 2023a, b).

Furthermore, pretreatment of complex biomass substrates remains a significant bottleneck, not only due to high energy and cost requirements for lignocellulose breakdown, but also because substrate inhibition can limit microbial efficiency during subsequent fermentation steps (Velvizhi et al. 2022). Continuous fermentation, on the other hand, maintains a steady state by continuously adding substrates and removing products, leading to higher productivity. Understanding fermentation processes in plant-based dairy alternatives can lead to the development of better-tasting and more eco-friendly alternatives. Recent advances in bioprocess optimisation have shown promise in addressing some of these challenges. Techniques such as fed-batch fermentation, which reduce substrate inhibition and enable high-density cell cultures, have improved yield and productivity (Huang et al. 2023a, b). Additionally, the development of genetically engineered microbial strains with enhanced tolerance to inhibitory conditions and improved metabolic pathways for LA production has further increased the viability of microbial fermentation on a commercial scale.

### Batch mode fermentation

**Fig. 3:**
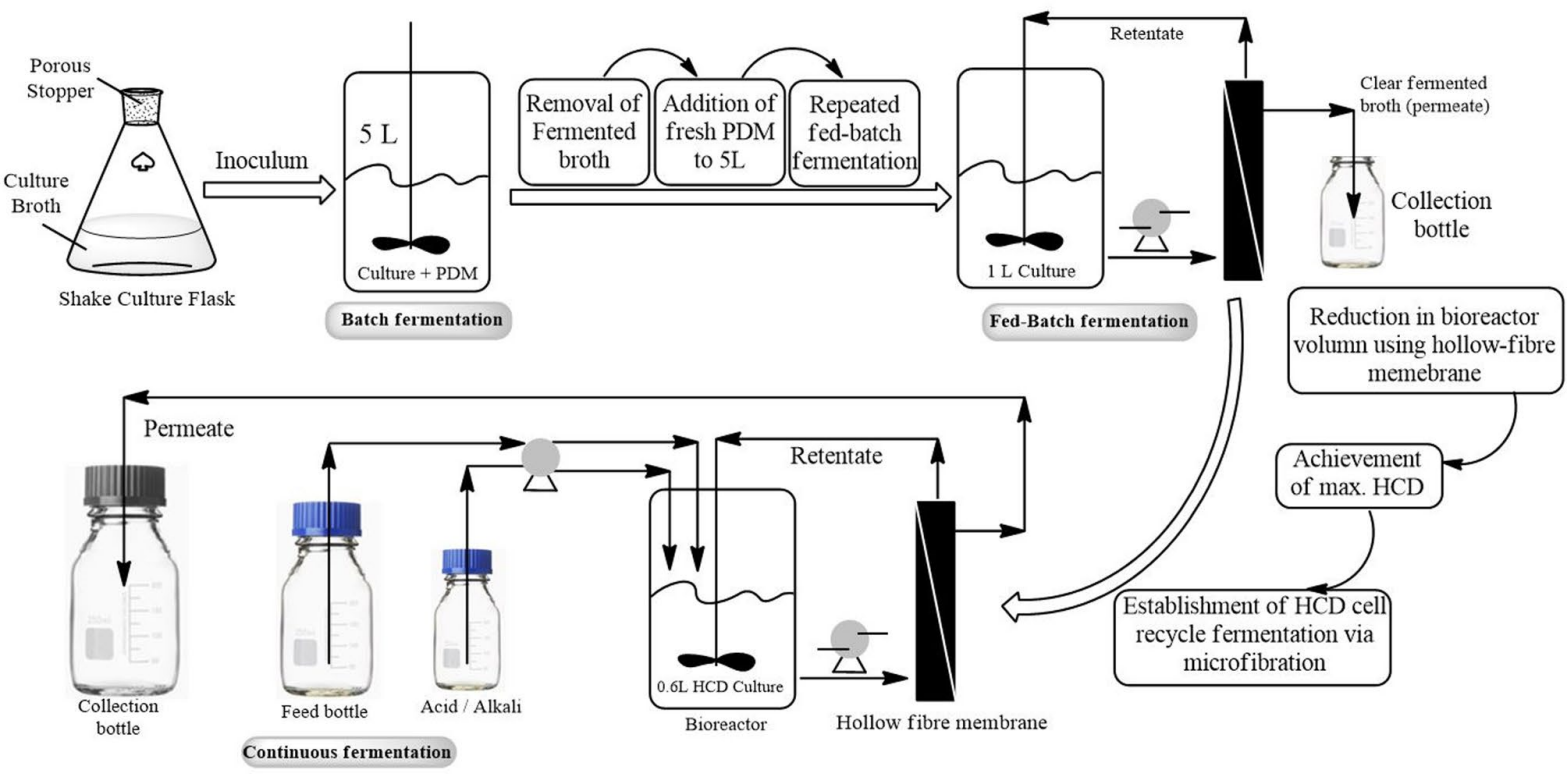
Fermentation modes description on a laboratory scale (Gupta et al. 2024)

Batch mode fermentation typically generates a high yield of LA, whereas the continuous system may enhance its productivity (Pleissner et al. 2016; Ren et al. 2022). In addition, batch fermentation is the most popular technique due to its ease of use, lower contamination levels, and higher titer. Batch fermentation involves the simultaneous addition of all substrates and nutrients to the bioreactor at the outset, allowing the fermentation to proceed without further input until it is complete. This method offers a high yield, as it can protect fermentation activity from inhibitors and increase the medium osmotic pressure and effects from fermentation byproducts. Additionally, batch fermentation is more straightforward to operate, making it suitable for small-scale production and laboratory settings. Its closed system design reduces the risk of contamination compared to continuous systems (Othman et al. 2017; Valério et al. 2024) (Fig. 3). However, according to Fan et al. (2017), batch fermentation is associated with several limitations, notably substrate inhibition and reduced productivity. The inhibition is largely attributed to the buildup of substrate and end products during the fermentation process (Rawoof et al. 2021). The accumulation of LA can hinder microbial activity and lower productivity, particularly when concentrations exceed certain thresholds (e.g., 100 g/L). Furthermore, once the substrate is depleted, the process must be restarted, resulting in reduced overall productivity compared to continuous fermentation.

### Continuous fermentation

Continuous fermentation involves the addition of fresh substrate while simultaneously removing an equal volume of culture broth, facilitating steady-state operation. This approach consistently yields high productivity rates as high as 10.34 g·L⁻¹·h⁻¹ using molasses, owing to the constant supply of nutrients and removal of inhibitory products (Olszewska-Widdrat et al. 2020). Continuous fermentation also mitigates product inhibition, as the continuous removal of LA helps maintain lower concentrations in the reactor, thereby enhancing microbial growth and metabolism. This method is often more scalable for large-scale industrial applications due to its efficient resource utilisation (Fan et al. 2017). Nevertheless, continuous fermentation has drawbacks, including a higher risk of contamination due to its open nature, the need for more sophisticated equipment, and the requirement for careful monitoring to maintain optimal conditions throughout the fermentation process. In short, continuous fermentation increases productivity and efficiency appropriate for industrial applications, whereas batch fermentation is beneficial for high yields in smaller operations. Table 1 below compares batch and continuous fermentation processes, while Table 2 shows the differences between microorganisms used in batch fermentation and continuous fermentation.

**Table 1:** Comparison of batch and continuous fermentation processes

Feature	Batch Fermentation	Continuous Fermentation
Yield	High initial yield	High productivity
Operation Complexity	Simple operation	More complex with advanced monitoring
Contamination Risk	Lower risk	Higher risk due to open system
Substrate Inhibition	Significant due to product accumulation	Reduced by continuous removal
Scalability	Suitable for small-scale	Better suited for large-scale production

**Table 2:** Comparison of biomass and LA production across microorganisms in batch vs. fed-batch cultures (Othman et al. 2017)

Microorganisms	Substrates	Biomass production in batch culture		Biomass production in fed-batch culture	
		Biomass production (g/L)	LA production (g/L)	Biomass production (g/L)	LA production (g/L)
*Sporolacto-bacillus nakayamae*	Sucrose	9	105	14	128
*Bacillus coagulans*	Glucose	18	-	25	-
*Lactobacillus rhamnosus B103*	Lactose and corn steep liquor	4.79	57	5.5	106
*Lactobacillus salivarius I 24*	Glucose	2.35	29.50	7.114	58.18
*Lactobacillus rhamnosus ATCC 10,863*	Molasses	3	16.5	5.2	22.0
*Lactobacillus plantarum LP02*	Glucose	2.53	-	10.12	-
*Lactococcus lactis WICC B-25*	Glucose	5.64	4.34	21.34	24.1
*Lactobacillus lactis*	Glucose	1.6	200	2.7	210

*LA: LA

## LA fermentation and resources ~ agriculture waste derived LA

LA fermentation is a bioprocess that converts carbohydrates into LA using microbial strains, primarily LA bacteria (LAB). The choice of resources, including substrates and microbial strains, plays a critical role in the efficiency and sustainability of LA production. Biomasses such as carbohydrates, starch, glucose, and lignocellulose offer a renewable and cost-effective source of carbohydrates for LA fermentation, serving as the primary source of LA production (Din et al. 2021; Kumar et al. 2020). Starch sources such as tapioca (Lian et al. 2023), potato, corn, rice, wheat, sweet potato and mung beans have also been reported for the production of LA (Neelam et al. 2012), while lignocellulosic materials used for the production of LA, such as corn stover (Parra-Ramírez et al. 2019), wastepaper (Yang et al. 2018), sugar beet pulp (Díaz et al. 2020) and bagasse (Darwin et al. 2018).

One of the significant challenges in LA production is the high cost associated with raw materials, which can account for a substantial portion of the total production expenses. Therefore, research has increasingly focused on finding inexpensive, sustainable alternatives. Studies have demonstrated that lignocellulosic biomass **(**Table 3**)**, a byproduct of agricultural and forestry activities, offers an abundant and low-cost raw material option. Utilising these byproducts reduces waste and lowers production costs, making the process more sustainable. However, the variability in substrate composition poses challenges in maintaining consistent fermentation performance.

**Table 3:** List of agricultural wastes and their chemical composition (Lee et al. 2014; Maraveas 2020)

Lignocellulosic Biomass	Composition (% w/w)		
	Cellulose	Hemicellulose	Lignin
Sugarcane Bagasse^a^	30.2	56.7	13.4
Rice Straw^a^	39.2	23.5	36.1
Corn Stalks^a^	61.2	19.3	6.9
Brewer spent grains^b^	24.5	23.8	15.8
Corncob^b^	45	35	15
Corn stover^b^	37.5	22.4	17.6
Corn fibers^b^	14.28	16.8	8.4
Hardwood stems^b^	40–55	24–40	18–25
Softwood stems^b^	40–50	25–35	25–35
Newspaper^b^	40–55	25–40	18–30
Wastepaper from chemical pulp^b^	60–70	10–20	5–10
Office paper	68.	12.4	11.3
Grasses^b^	25–40	35–50	10–30
Switchgrass^b^	31–45	20.4–31.4	12-17.6
Coastal bermudagrass^b^	25	37.5	6.4
Leaves^b^	15–20	80–85	0
Wheat straw^b^	30	50	15
Cottonseed hairs^b^	80–95	5–20	0
Nutshells^b^	25–30	25–30	30–40
Pinewood^b^	46.4	8.8	29.4
Sugarcane bagasse^b^	40–50	25–35	17–20

Lignocellulosic biomass comprises primarily cellulose and lignin. Cellulose, a linear polymer consisting of D-pyranose glucose units linked *via* β-1,4-glycosidic bonds, imparts rigidity and structural stability to the primary cell wall. In contrast, lignin is a complex phenolic polymer synthesised through the oxidative coupling of p-hydroxycinnamyl alcohols, specifically p-coumaryl, coniferyl, and sinapyl monolignols, which collectively form a protective matrix shielding plant tissues from pathogenic invasion (Hernández-Beltrán et al. 2019). Although the lignocellulosic biomass is less expensive than carbohydrates, the byproducts such as furfural, hydroxymethyl furfural (HMF), syringaldehyde and vanillin (which are obtained during the lignocellulosic pretreatment phase), inhibit the microbial growth and lower the production of LA. However, pretreating lignocellulosic biomass, such as through physical, chemical, biological and mechanical methods, increases the purification cost (Din et al. 2021).

Acidic and chemical residues from lignocellulosic pretreatments cause environmental damage due to incompetent waste management and inhibit microbial growth during fermentation; thus, detoxification and neutralising agents are required after pretreatments. The processes are expected to incur an additional cost for the production of LA (Shan et al. 2023). Genetically modified microbial such as *Saccharomyces cerevisiae* BTCC3, BTCC LX1, BTCC3 LX5, BTCC3 LA1, BTCC3 LA15 and BTCC3 LA2 with exogenous L-LDH genes to enable the fermentation of LA from glucose are robust strains which are survived at low pH and tolerance with lignocellulosic-derived chemical inhibitors, including acetic acid, formic acid, furfural and levulinic acid. A few genetically engineered *S. cerevisiae* strains are used in the production of LA using glucose as the substrate (Sornlek et al. 2022). The yield of LA obtained from *S. cerevisiae* hybrid2 (1.54 g/L/h) was compared to different types of engineered *S. cerevisiae*, such as *S. cerevisiae* OC2 (1.21 g/L/h), *S. cerevisiae* SR8 (1.05 g/L/h) and *S. cerevisiae* JHY5330 (0.41 g/L/h). *S. cerevisiae* hybrid2 consumed glucose efficiently compared to CEN.PK2_DLDH∆gpd during the SSF. Genetically modified strains, such as *Saccharomyces cerevisiae*, play a crucial role in improving LA production efficiency through strain engineering techniques. By integrating targeted genetic modifications, researchers enhance metabolic pathways, substrate utilisation and yield optimisation, making fermentation processes more sustainable and economically viable (Choi et al. 2024). For LA production, strain engineering is generally favored as it enables direct enhancement of microbial robustness and metabolic efficiency under acidic conditions, thereby supporting high yields and cost-effective industrial-scale fermentation. In contrast, process optimisation alone is often considered less effective, as it primarily modifies external parameters without addressing inherent biological limitations of the production strain.

*Bacillus* sp. P38 was used in batch fermentation for the production of L-LA. Peng et al. (2013) reported that *Bacillus* sp. P38 is a promising producer of 100% L-LA from cellulosic hydrolysate (Peng et al. 2013). The strain produced 180 g/L L-LA in fed-batch fermentation. It showed a strong tolerance capacity to 2-furfural (up to 10 g/L) and 6 g/L vanillin or acetic acid. Other researchers proved that *Pediococcus acidalactici* XH11 can produce 100% D-LA using undetoxified acid-pretreated corncob slurry (Shan et al. 2023). Another study on lignocellulosic biomass was conducted for LA production using *Bacillus amyloliquefaciens* to ferment sugarcane molasses, further supporting the viability of these byproducts for fermentation (Ajala et al. 2020). Considering that sugarcane molasses contains hazardous substances, a new microbial consortium CEE-DL15, converted sugarcane molasses to LA was evaluated which consisted of *Clostridium sensustricto* (57.29%), *Escherichia* (34.22%), and *Enterococcus* (5.32%) to yield LA 0.81 g/g and maximum productivity of 4.49 g/(L.h) (Sun et al. 2017).

The pretreatment with dilute ethylenediamine and simultaneous saccharification and fermentation (SSF) of rice straw effectively produces second-generation L-LA to avoid using large amounts of inorganic alkali in lignocellulose pretreatment (Chen et al. 2019). The study reported that in fed-batch SSF under non-sterilised conditions, a maximum of 63.5 ± 3.0 g L^− 1^ and 296.8 g Kg^− 1^ L-LA was produced from raw rice straw using *Bacillus coagulans* LA-15-2. Additionally, the final L-LA concentration reached 92.5 g L^− 1^. A year later, another second-generation LA derived from rice straw pretreated with imidazolium ionic liquid [EMIM][OAc] and subsequently fermented with a promising *Lactobacillus plantarum SKL-22*, with a yield of 36.75 g/L (Yadav et al. 2021). Fermentation of corn stalks using *Pediococcus acidilactici* has yielded promising results in producing high-titer LA through fed-batch simultaneous saccharification and fermentation (SSF). A higher yield of LA was achieved up to 0.77 g/g of corn stover with a titer of 92.01 g/L when corn stover was pretreated (Wang et al. 2015).

Emerging evidence highlights the anaerobic ensiling of agricultural dry straw residues as an innovative pretreatment approach enabling the targeted conversion of lignocellulosic biomass into bio-based chemicals(Lillington et al. 2020). *B. coagulans*, known for its robustness, has been employed in a simultaneous saccharification and fermentation process. For instance, it produced LA with a yield of 0.86 g/g from corn stalks in a hybrid fermentation process, while simultaneously generating ethanol (Yang et al. 2018).

## Applications of LA

**Fig. 4:**
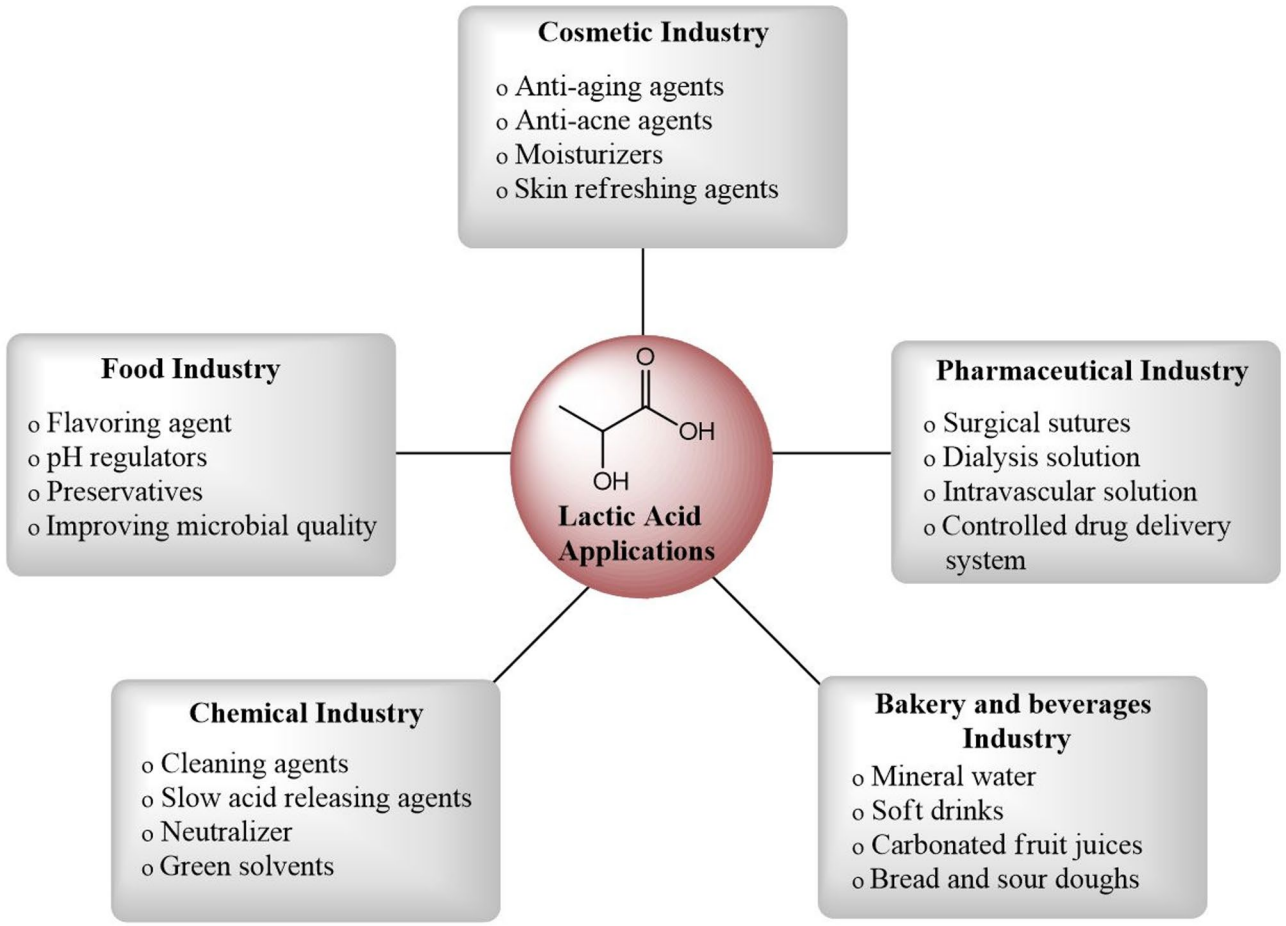
General applications of LAs

From a bio- and circular economy standpoint, LA made from agro-food waste can enhance process sustainability, waste reduction and residual biomass valorisation (Costa et al. 2021). Cutting-edge fermentative methods for producing LA from agricultural waste can potentially reduce production costs and environmental impact, thereby benefiting several businesses and addressing environmental issues (Huang et al. 2021). LA derived from agricultural waste holds promise for the production of biodegradable and biocompatible polymers, offering potential for further applications. The integration of innovative pretreatment methods, such as hydrolysis and fermentation techniques, coupled with supportive policy initiatives, can render LA production from agricultural waste streams competitive and sustainable (Djukić-Vuković et al. 2019) (Fig. 4).

Valorisation of agricultural waste-derived LA offers cross-sectoral applications in the cosmetic, culinary, pharmaceutical, and textile industries. Concurrently, the upcycling of underutilised agro-industrial residues into LA via microbial fermentation aligns with global demands for sustainable bioproduction, addressing food/feed security and environmental sustainability through cost-effective resource utilisation (Panesar and Kaur 2015).

The food, pharmaceutical, and chemical sectors have the potential to increase their use of LA due to its high added value, safety, and ability to be produced from various waste products. The food and pharmaceutical industries favour the isomer L (+) (Castillo Martinez et al. 2013). Probiotics utilise the metabolic properties of LA bacteria to support human health. Additionally, the food industry utilises these bacteria to enhance flavour profiles, improve nutritional value, reduce harmful compounds and extend product shelf life (Wang et al. 2021). Different biopolymers produced by LA bacteria strains can be applied in new ways, such as in the production of dairy products, low-fat cheeses, sourdough bread, and certain drinks in the fermented food sector (Torino et al. 2015). LAB is important to the functional food sector due to its probiotic qualities and ability to produce a variety of physiologically active metabolites that enhance the nutraceutical qualities of food products (Abedin et al. 2024). Given its wide range of uses across multiple industries, LA has been utilised in many processed foods, including baked goods, dairy products, and soft drinks, as an acidulant, preservative, flavouring, or buffering agent in the food business. In terms of food additives, the US Food and Drug Administration (FDA) and other regulatory bodies have designated it as GRAS (generally recognised as safe) (Ajala et al. 2020). Sourdoughs, fermented dough and several types of bread are examples of LA applications. The application of LA fermentation enhances the quality of the bread loaf, particularly by improving the coloration of the crust. Bread goods have a longer shelf life when supplemented with LA because it prevents the growth of mould and rope.

Calcium and sodium stearoyl lactates are effective emulsifiers in the baking industry, allowing for reduced baking times and improved product quality. These compounds serve as effective emulsifiers and conditioners for yeast-leavened baked goods while stabilising butter and acting as substitutes for egg albumin (Kim et al. 2022). The food industry uses about 70% of LA to create yoghurt and cheese. The primary byproduct of co-fermenting *Lactobacillus bulgaricus* and *Streptococcus thermophilus* is yoghurt. Casein micelles aggregate during the cheese-making process due to a drop in pH resulting from LA emission. Depending on the sensory qualities required in the finished product, direct acidification with LA may reduce the possibility of unfavourable microbes proliferating (Castillo Martinez et al. 2013).

LA derivatives, including lactate esters, are frequently employed due to their emulsifying and hygroscopic qualities. It is a supplement used in the pharmaceutical sector to synthesise anti-osteoporosis and dermatology medications (Castillo Martinez et al. 2013). Specifically, antiacne solutions, topical ointments, parental solutions, lotions, humectants, dialysis applications, and anticaries agents are among the pharmaceutical formulations that use LA. In addition, LA is utilised as a dialysis solution in traditional artificial kidney devices and as an electrolyte in various intravenous fluids to replenish bodily fluids. Moreover, LA is frequently utilised in various mineral products, including surgical sutures, prostheses, and tablets. For calcium shortage and as an anti-inflammatory drug in dental treatments, the calcium salt of LA is widely utilised. Medications with an LA base have antibacterial and anticancer properties. LA is a common ingredient in sanitiser formulations and the management of skin conditions, including warts (Kim et al. 2022).

LA is utilised as a building block molecule in the chemical industry for processes such as oil-free plastic replacement and biodegradable polymers (Alves de Oliveira et al. 2018). LA has gained popularity as a monomer for biodegradable PLA (Augustiniene et al. 2022; Wee et al. 2006). A promising biomedical material for medication administration, implants, and tissue engineering, PLA is an environmentally benign, biodegradable polymer with good biocompatibility and biodegradability. LA undergoes direct condensation polymerisation to yield high-molecular-weight polylactic acid that is strong and durable enough to be processed into films and moulded goods (Ajioka et al. 1995; Ramezani Dana and Ebrahimi 2023). PLA is a possible biobased substitute for petroleum-based polymers in various industries because it can be blended with other polymers to customise its properties and avoid restrictions on its uses (Nofar et al. 2019). The L(+) of LA and its salts exhibit high biocompatibility in the human body, making them valuable in pharmaceutical applications. These include their use as electrolytes in parenteral/intravenous (I.V.) solutions, substitutes for acetate in dialysis formulations (due to their reduced adverse effects), components of drug-release matrices, and active ingredients in topical formulations for skin hydration. Chiral LA is well-accepted by pharmaceutical manufacturers due to its low cost, non-toxic and synthetically versatile nature (Abd Alsaheb et al. 2015).

LA has been widely used in cosmetics to produce aesthetic goods and dental hygiene products. This is due to the moisturising, antibacterial, and rejuvenating properties of LA for the skin. Skin problems can be improved, and diseases can be avoided with the help of LA bacteria and their extracts. Applying topical LA helps reduce melanin pigment, resulting in brighter skin, and reduces brown spots or smudges, alleviating or removing skin discolouration (Abd Alsaheb et al. 2015). It also reduces surface roughness and moderate wrinkles caused by environmental photo-damage (Huang et al. 2020). In addition to its antibacterial properties, LA is commonly used as a moisturiser, pH regulator and skin-lightening agent. It diminishes corneocyte cohesion immediately above the granular layer by detaching and desquamating the stratum corneum. LA can also treat acne, scars, age spots, and seborrhea by increasing the synthesis of glycosaminoglycans and thickening skin (Komesu et al. 2017). Tyrosinase activity is inhibited by LA, which prevents skin from browning. It substitutes glycolic acid, an anti-ageing substance that minimises sun damage and softens wrinkles. Because LA increases sensitivity to UV light, care must be exercised while supplementing cosmetic creams with LA (Kim et al. 2022).

## Overcoming challenges in LA production

LA offers significant advantages as it can be produced from a diverse range of waste materials, thereby enhancing its added value while ensuring safety. Its applications span multiple sectors, including chemicals, food, and pharmaceuticals, with considerable growth potential. However, challenges persist in the manufacturing process, particularly in the purification and recovery of the product (Castillo Martinez et al. 2013). Furthermore, improving fermentation technology remains a challenge that requires further study and advancement to increase the yield and efficiency of LA production. Resolving these issues is essential to maximising the advantages of LA across a range of sectors.

Microbial fermentation of LA can offer higher efficiency and reduced production costs. Nevertheless, issues, including feedstock costs, energy usage, inhibitory compounds and the preservation of optical purity persist (Huang et al. 2023a). The microbial synthesis of optically pure LA faces limitations. Nevertheless, advances in fermentation technology and metabolic engineering can overcome these hurdles, enabling the economical and sustainable production of biodegradable plastics (Abdel-Rahman and Sonomoto 2016; Acedos et al. 2022). High manufacturing costs and the need for improved pretreatment, enzyme hydrolysis, and fermentative technologies are among the obstacles to the production of LA. Despite the environmental benefits and productivity gains of manufacturing LA from renewable resources, its widespread adoption remains limited by high production costs and the need for improved pretreatment, enzyme hydrolysis, and fermentation technologies(Ahmad et al. 2020). Reducing production costs through recombinant cellulolysis, enhancing microwave-assisted deep eutectic solvent pretreatment and overcoming feedback-, substrate- and end-product inhibition through continuous simultaneous saccharification and fermentation are other challenges in producing LA. While microwave-assisted deep eutectic solvent pretreatment provides quick and safe byproducts, the recombinant cellulolytic method in LA bacteria may lower production expenses (Ajala et al. 2020). Cost-effective biomass is a potential way to lower the cost of substrates, which is the primary obstacle to LA bioproduction. Recent developments in the bioproduction of PLA from renewable resources offer potential for lowering manufacturing costs and resolving issues with efficient production (Huang et al. 2023a).

Other challenges in the production of LA include the limited supply of traditional fermentable starchy substrates and the need for accessible and affordable alternatives. Lignocellulosic biomass, comprising agricultural and forest residues, offers a promising solution due to its abundant supply and low cost. Nevertheless, several barriers exist to its utilisation, including the complexity of its polymeric structure, which necessitates advanced pretreatment methods to release fermentable sugars and effective detoxification strategies to mitigate inhibitory compounds (Nwamba et al. 2021). Additionally, the development of efficient microbial strains, innovative separation techniques, and process optimisation is crucial for enhancing LA production from lignocellulosic feedstocks. Addressing these challenges will be crucial for harnessing lignocellulosic biomass as a sustainable feedstock for LA production, thereby contributing to a circular bioeconomy and increasing the availability of value-added compounds for various industries (Yankov 2022).

The challenges associated with LA production often overlap with fermentation techniques, particularly in terms of microbial efficiency, substrate utilisation and downstream processing. These issues influence both process scalability and economic feasibility (Huang et al. 2023b). Achieving improved yield, productivity and optical purity in LA production while utilising inexpensive resources remains another significant challenge. New approaches include minimising harmful chemicals and making effective use of mixed sugars. Microbial fermentation offers a promising solution by producing optically pure LA with minimal harmful chemicals and effectively utilising mixed sugars (Rawoof et al. 2021). However, several obstacles persist, including high substrate and pretreatment costs, medium expenses, and the risk of contamination due to neutral pH and mesophilic conditions. Innovative techniques (i.e., low-cost organic waste as feedstocks) have shown potential in reducing expenses and enhancing output. For instance, *Enterococcus durans* BP130 has been used to generate LA from various organic wastes efficiently, thus highlighting the potential for cost-effective and sustainable production methods (Hassan et al. 2019). Addressing these challenges through novel fermentation strategies and optimisation techniques is crucial for overcoming current limitations and improving the efficiency of LA production.

## Recent advances in LA

Utilising renewable resources, cutting costs, and increasing yield are the main goals of recent developments in the manufacture of LA. The use of low-cost substrates, innovative fermentation methods and genetic and metabolic engineering are important advancements. Recent developments in technology, recovery, and LA manufacturing have increased yield, decreased production costs, and allowed the green chemical industry to create eco-friendly goods using cutting-edge methods, including simultaneous saccharification and cell recycling systems (Kim et al. 2022). Recent developments in LA generation include cell recycle systems, immobilisation methods, genetic and metabolic engineering, and simultaneous saccharification. The food and life sciences sectors are poised to benefit from recent advancements in LA production, which utilise genetic and metabolic engineering to enhance yield and reduce costs (Eş et al. 2018).

The current state of LA production involves genetically modifying microbial strains to produce LA from unconventional carbon sources and modifying bacteria to withstand acidic environments. To manufacture LA from non-traditional carbon sources, metabolic engineering of microbial strains is required. This will lower production costs and improve the utilisation of agricultural biomass for products with added value (Juturu and Wu 2016). Implementing inexpensive, low-cost raw materials, improving the utilisation of inexpensive, low-cost carbon and nitrogen sources and employing effective strain modification technologies are recent developments in LA production that have lowered production costs and aided in the growth of the LA business (Tian et al. 2021).

Recent developments in LA manufacturing include improved fermentation techniques, low-cost substrates such as dairy products, food waste, and the enhancement of algal biomass output through immobilisation and cell-recycling techniques. The production and productivity of biodegradable PLA materials can be increased, and LA production costs can be significantly decreased by utilising low-cost substrates and sophisticated fermentation techniques (Abdel-Rahman et al. 2013). High-yield productivity employing affordable, readily available substrates and integrated biorefinery platforms for waste materials are examples of recent developments in the manufacture of LA. Although the manufacture of LA from renewable resources offers significant environmental benefits and increases productivity, its widespread use is limited by high manufacturing costs (Ahmad et al. 2020).

LA production has advanced recently, with several applications across multiple industries and efficient synthesis from low-cost raw materials. Environmental concerns and the scarcity of petrochemical feedstocks have sparked interest in the biotechnological fermentation of LA, which has promise for future uses and financial benefits (Wee et al. 2006). Recent research has demonstrated that employing renewable resources, such as acid whey, coffee mucilage, and rice husk hydrolysate, can significantly reduce production costs and contribute to the development of biodegradable plastics. D-LA is essential for the creation of heat-resistant PLA (Alexandri et al. 2019). The generation of optically pure LA through fermentation has expanded, emphasising both metabolically modified strains and natural microorganisms. Recent developments in the biotechnological synthesis of LA from renewable resources hold promise for cost-effectiveness and environmental friendliness, with applications in food, medicine, cosmetics, and biodegradable polymers (Wang et al. 2018).

Recent developments in LA manufacturing aim to reduce costs, increase yield, and utilise renewable resources. These advancements include the use of low-cost substrates, innovative fermentation methods, and metabolic engineering. Combined with genetic and metabolic engineering, these methods have led to the development of eco-friendly products and reduced production costs. The use of low-cost substrates and advanced fermentation techniques has also contributed to the growth of the LA industry. Agricultural waste can be transformed into LA, a biodegradable polymer with potential applications in various industries. The production of LA from agricultural waste can reduce production costs and environmental impact, benefiting various businesses. The food, pharmaceutical, and chemical sectors can benefit from LA’s high added value, safety, and versatility. The food industry uses LA in various products, such as dairy products, yoghurt, and cheese. In the pharmaceutical sector, LA is used in medications for anti-osteoporosis and dermatology, as well as in dialysis solutions and intravenous fluids. In the chemical industry, LA is used as a building block for biodegradable polymers, such as PLA, a promising biomedical material for medication administration, implants, and tissue engineering. LA is also used in cosmetics for its moisturising, antibacterial, and rejuvenating properties.

## Conclusion

In conclusion, LA production from agricultural waste provides a sustainable and eco-friendly alternative to traditional methods by utilising renewable feedstocks such as sugarcane bagasse, rice husk, and corn stover. This approach not only reduces waste but also provides a cost-effective solution for industries that are dependent on LA, such as food, pharmaceuticals and biodegradable plastics. Despite challenges such as substrate variability and contamination, advancements in fermentation techniques, including batch and continuous fermentation, as well as the genetic engineering of microbial strains like Lactobacillus spp. and Rhizopus spp., have improved the efficiency and yield of LA. Integrating novel strategies, such as consolidated bio-saccharification and efficient producers like *Geobacillus stearothermophilus*, further enhances the potential for industrial-scale production. As LA continues to play a crucial role in various sectors, ongoing research and development will be essential for optimising production processes, ensuring sustainability and expanding its applications in a circular economy. LA’s applications span multiple industries, reflecting its high added value, safety, and versatility.

## Electronic supplementary material

Below is the link to the electronic supplementary material.

Supplementary Material 1

Supplementary Material 2

## Data Availability

Data sharing does not apply to this article, as no datasets were generated or analysed during the current study.
