# Supplementary material for: Sustainable lactic acid production from agricultural waste: a review of current techniques, challenges and future directions
Source: Bioresour Bioprocess. 2025 Jul 29;12(1):81. doi: 10.1186/s40643-025-00923-3 (PMC12307837; doi:10.1186/s40643-025-00923-3)
Supplement: Supplementary file 1 — Supplementary Material 1 [file 40643_2025_923_MOESM1_ESM.docx]

Respected Editor,

The comments raised by the reviewers are addressed in the tables below. Revisions have been made to the original manuscript and changes are highlighted using track changes mode in MS Word.

**Decision on Manuscript: ‘**Sustainable Lactic Acid Production from Agricultural Waste: A Review of Current Techniques, Challenges and Future Directions’

| **Reviewers' comments:** | **Author’s Response** |
| --- | --- |
| **Journal Comments**   1. We notice that Figure 3 has been referenced in the main text of your manuscript file but this figure has not been included with your submission. Please either upload this figure as a separate 'Figure' file and include a corresponding legend in the main manuscript file or update the references in the main text of your manuscript accordingly.   2. We notice that figure 4-5 has not been referenced in the main text of your manuscript file. If figures are not cited in the manuscript they will not appear in the html (online) version if your paper is accepted for publication. Therefore, it is essential they are mentioned at least once in the text and, we strongly recommend, in the order in which they are numbered.  3. The graphical abstract should be 920 x 300 pixels and a maximum of 150KB jpeg, png or svg file for better resolution. Please change file type. | Thank you for reviewing my draft.   1. Figure 3 is actually merged in Figure 2. The Figure numbering has been updated as a separate 'Figure' file’ with inclusion of a corresponding legend in the main manuscript file. 2. Figures citation inside text of manuscript has been inserted and carefully cross checked. 3. GA resolution and file size has been updated. File type is Jpeg inserted. |
