# Supplementary figures and images for: Sustainable lactic acid production from agricultural waste: a review of current techniques, challenges and future directions
Source: Bioresour Bioprocess. 2025 Jul 29;12(1):81. doi: 10.1186/s40643-025-00923-3 (PMC12307837; doi:10.1186/s40643-025-00923-3)

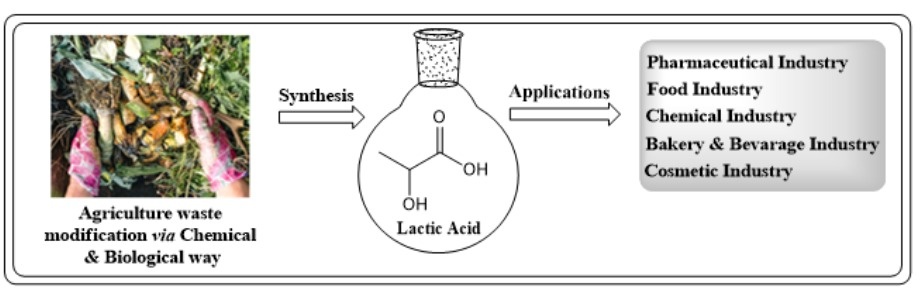

Supplement: Supplementary file 2 — Supplementary Material 2 [file 40643_2025_923_MOESM2_ESM.jpg]
